# Supplementary material for: Betapapillomaviruses in p16-Negative Vulvar Intraepithelial Lesions Associated with Squamous Cell Carcinoma
Source: Viruses. 2023 Sep 19;15(9):1950. doi: 10.3390/v15091950 (PMC10537070; doi:10.3390/v15091950)
Supplement: Supplementary file 1 [file viruses-15-01950-s001.zip › Supplemental Materials.pdf]

Supplemental Materials

Supplemental Data S1. Immunohistochemistry protocols.

Deparaffinization was carried out on the instrument, as was heat-induced epitope retrieval in the form of "cell conditioning" with CC1 buffer (Ventana, #950-224), an EDTA based buffer pH 8.4, for 62 minutes at 95◦C. Then, 100:l primary antibody **p53** (RTU) was applied for 16 min at 37 degrees Celsius, the slide was rinsed with reaction buffer (Ventana, #950-300) and discovery OmniMap anti-Mouse HRP (Ventana, #760-4310) and incubated for 16 min at 37 degrees Celsius. The slide was then rinsed with Reaction Buffer (Ventana, #950-300) followed by discovery ChromoMap DAB detection (Ventana, #760-159) was applied for the preset time. Slide removed from the instrument and rinsed with dawn dish soap and warm tap water. Rinsed with dH_2O. Slide was counterstained with Harris hematoxylin (VWR, #10143-606) diluted 1:5 with dH_2O for 45 seconds. Rinsed with dH_20. Dehydrated. Dipped in Xylene. Coverslipped using Mounting Medium (Thermo, Cat# 4112).

Supplemental Data S2. HPV Genotyping using the Luminex technology.

The multiplex type specific PCR method uses specific primers for the detection of 21 high-risk α-HPV types (6, 11, 16, 18, 26, 31, 33, 35, 39, 45, 51, 52, 53, 56, 58, 59, 66, 68, 70, 73, 82); 46 β-HPVs from β-species 1 (5, 8, 12, 14, 19, 20, 21, 24, 25, 36, 47, 93, 98, 99, 105, 118, 124, 143, and 152), β-species 2 (9, 15, 17, 22, 23, 37, 38, 80, 100, 104, 107, 110, 111, 113, 120, 122, 145, 151, 159, and 174), β-species 3 (49, 75, 76, and 115), β-species 4 (92), and β-species 5 (96 and 150); and 52 γ-HPVs from γ-species 1 (4, 65, 95, and 173), γ-species 2 (48 and 200), γ-species 3 (50), γ-species 4 (156), γ-species 5 (60, 88), γ-species 6 (101, 103, and 108), γ-species 7 (109, 123, 134, 149, and 170), γ-species 8 (112, 119, 164, and 168), γ-species 9 (116 and 129), γ-species 10 (121, 130, 133, and 180), γ-species 11 (126, 169, 171, and 202), γ-species 12 (127, 132, 148, 165, and 199), γ-species 13 (128), γ-species 14 (131), γ-species 15 (179), γ-species 18 (156), γ-species 19 (161, 162, and 166), γ-species 20 (163), γ-species 21 (167), γ-species 22 (172), γ-species 23 (175), γ-species 24 (178 and 197), γ-species 25 (184), and γ-species 27 (201), and SD2. HPV-SD2 official classification is pending and it was not included in any species (48). Two primers for the amplification of the β-globin gene were included to provide a positive control for the quality of the DNA in the sample. Following multiplex PCR amplification, 10 µL of each reaction mixture was analyzed by multiplex genotyping using the Luminex technology as previously described (30,39). Results were expressed as the median fluorescence intensity (MFI)

of at least 100 beads per bead set. For each probe, MFIs with no respective PCR product added to the hybridization mixture were considered background values. The cutoff was computed by adding 5 MFI to 1.1 times the median background value.

Supplemental Table S1. Primer and probe set for HPV 5, 9, 24 and 111.

HPV type	forward primer	reverse primer	probe	Reference
5	GGCTGGAGCACTAAAAGATG	CATTGATCTGTGCCAATACCT	GATATTTCATATTCTTCTACATGTCTTTGATA	[39]
9	GGGTTGGAACATCAGGTCAC	GCCTGTCATCCATTGTTGTG	GGTTAGAGACACAGAAAACTCTAGCA	/
24	GGAAGTAGCTGAGAGGTGTG	GATCTACTTTGTTGTAGTGTTC	ACCATCTTGAATTACTGAATTTACTAACTT	[39]
111	GCAAGTCAAAATGTTTATAATAGGATGTG	GCTGGTTTCCTGCATCATCA	TATGGGAGAATACTGGGACAAAGCAAAACCCT	[43]

Supplemental Data S3. Quantitative PCR standard curves and calculations.

Supplemental Data S4. RNA In Situ Hybridization

RNA ISH was performed on FFPE tissue from selected cases with identifiable β-HPV and sufficient tissue for additional analysis. Altogether, 11/26 (42.3%) cases were stained for β-HPV RNA ISH. ISH for HPV E6/E7 transcript was completed using RNAscope (2.5 HD Reagent Kit-Brown, 322300, Advanced Cell Diagnostics, Newark, CA, USA) with probes specific for HPV types 5, 9, 24 and 111 (probe 421581, 421601, 421701 1121931-C1, respectively) according to the manufacturer's instructions. Stains were interpreted by a gynecologic pathologist (MBF). A negative control probe was included with each sample. No HPV RNA was detected in any of the samples.

Supplemental Table S2. Clinicopathologic characteristics of enrolled patients.

**Characteristic**	**All patients N = 26**		**p16 positive* N = 14**		**p16 negative N = 12**	
Mean age at diagnosis (years)	62,1	(31-88)	56.6	(31-80)	68.5	(40-88)
Ethnicity						

white	25	(96.2%)	13	(92.9%)	12	(100%)
native American	1	(3.8%)	1	(7.1%)	0	(0%)
Smoking status						
current or former	13	(50%)	8	(57.1%)	5	(41.7%)
never	13	(50%)	6	(42.9%)	7	(58.3%)
Immunosupression						
Yes	3	(11.5%)	3	(21.4%)	0	(0%)
No	23	(88.5%)	11	(78.6%)	12	(100%)
Lichen Sclerosus history						
Yes	1	(3.8%)	1	(7.1%)	0	(0%)
No	25	(96.2%)	13	(92.9%)	12	(100%)
Diagnosed with invasive SCC						
Yes	26	(100%)	14	(100%)	12	(100%)

*one case showed focal p16 overexpression, and null type pattern

Supplemental Table S3. Clinicopathologic characteristics of patients with atypical squamous proliferations (ASP) and high-grade squamous intraepithelial lesions (HSIL), compared by Fisher's exact test.

**Characteristic**	**ASP n=9**		**HSIL n=14**		**p value**
Mean age at diagnosis (years)	68.1	(40-88)	56.5	(31-80)	0.085
Ethnicity					
white	9	(100%)	13	(92.9%)	0.583
native American	0	(/)	1	(7.1%)	
Smoking status					
current or former	5	(60.0%)	8	(57.1%)	0.143
never	4	(40.0%)	6	(42.9%)	
Immunosupression					
Yes	0	(/)	3	(21.4%)	0.098
No	9	(100%)	11	(78.6%)	
Diagnosed with invasive SCC					
Yes	9	(90.0%)	14	(100%)	0.67

Supplemental Table S4. MFI levels of β-HPV DNA in patients with detectable β-HPV in intraepithelial vulvar lesions.

**Patient number**	**β-HPV**	**MFI**	**β-HPV**	**MFI**
5	HPV111	4		
6	HPV105	19		

10	HPV24	9		
11	HPV5	2		
15	HPV5	5	HPV23	19
20	HPV75	58	HPV145	116
24	HPV36	134	HPV25	60
28	HPV111	3		
45	HPV9	26		
50	HPV9	46	HPV38	46
57	HPV110	231		

Supplemental Figure S1. High-risk HPV RNA in situ hybridization was positive (white arrow) in Case 57 (p16-negative ASP with detectable HPV 16 and HPV 110 as per HPV genotyping).

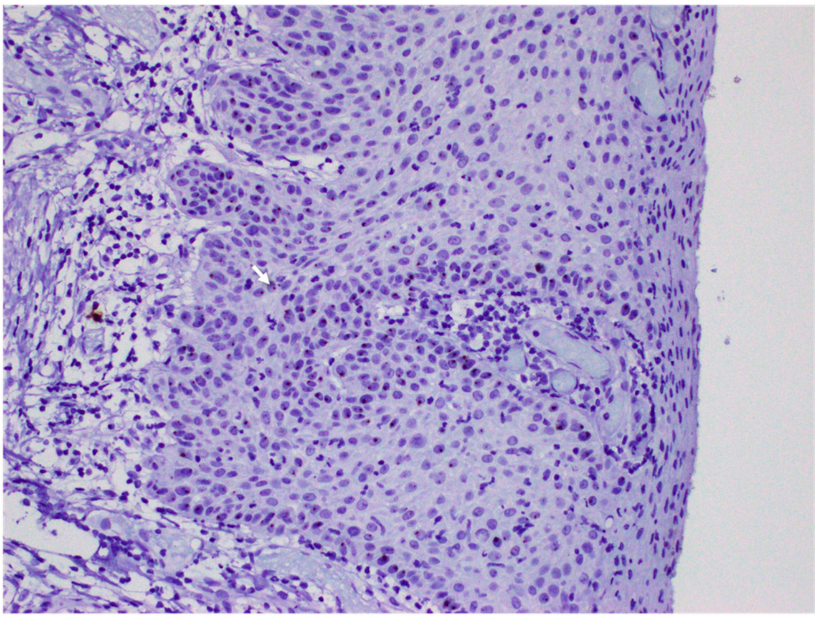

Table S5. Overview of morphology, immunohistochemistry, HPV genotyping, and interobserver variability analysis.

Morphology	Patient Number	IHC		Disagreement p53, any	Disagreement dg, any	HPV Type Summary					
		p16	p53			β		γ	Low-Risk α	High-Risk α	
ASP	3	N	WT	No	No					HPV 16	
	5	N	OB	/	/	HPV 111					
	6	N	OB	Yes	No	HPV 105				HPV 16	
	11	N	OB	Yes, OV vs. MT	Yes, ASP vs. DVIN	HPV 5				HPV 31	HPV 51
	15	N	WT	Yes, OB vs. MT	Yes, ASP vs. DVIN	HPV 5	HPV 23				

	28	N	WT	No	No	HPV 111				HPV 31	HPV 51
	42	N	OB	Yes, OB vs. WT	Yes, ASP vs. DVIN						
	45	N	OB	Yes, OB vs. WT	No	HPV 9				HPV 16	
	57	N	OB	/	/	HPV 110				HPV 16	
	7	N	OV	No	No						
DVIN	47	N	OV	Yes, OV vs. OB vs. MT	No						
	10	N	OV	No	No	HPV 24					
	20	P	BS	No	No	HPV 75	HPV 145			HPV 16	
	21	P	BS	No	No					HPV 16	HPV 31
	22	P	BS	/	/						
	24	P	BS	No	No	HPV 36	HPV 25	HPV 130, 132		HPV 16	HPV 39
	26	P	OV	Yes, OV vs. WT vs. MT	Yes, HSIL vs. DVIN					HPV 16	HPV 31
	31	P	WT	No	No					HPV 18	
HSIL	32	P	BS	No	No					HPV 16	
	34	P	BS	No	No						
	36	P	OB	Yes, WT vs. MT	No				HPV 6	HPV 16	HPV 31
	40	P	OB	Yes, OB vs. MT	Yes, HSIL vs. DVIN				HPV 6	HPV 16	
	49	P	BS	No	No					HPV 31	HPV 56
	50	P	BS	Yes, WT vs. MT	No	HPV 9	HPV 38			HPV 16	
	51	P	BS	No	No					HPV 16	
	56	P	BS	No	No					HPV 16	

OB, overexpressed basal; OV, mutant overexpression, basal/parabasal; WT, wild type; MT, mutant.
